# Novel C-2 Symmetric Molecules as α-Glucosidase and α-Amylase Inhibitors: Design, Synthesis, Kinetic Evaluation, Molecular Docking and Pharmacokinetics

**DOI:** 10.3390/molecules24081511

**Published:** 2019-04-17

**Authors:** Danish Shahzad, Aamer Saeed, Fayaz Ali Larik, Pervaiz Ali Channar, Qamar Abbas, Mohamed F. Alajmi, M. Ifzan Arshad, Mauricio F. Erben, Mubashir Hassan, Hussain Raza, Sung-Yum Seo, Hesham R. El-Seedi

**Affiliations:** 1 Department of Chemistry, Quaid-I-Azam University, Islamabad 45320, Pakistan; danish.scientist.shahzad@gmail.com (D.S.); fayazali@chem.qau.edu.pk (F.A.L.); mrpervaiz@gmail.com (P.A.C.); ifzan_ifzan@yahoo.com (M.I.A.); 2 Department of Physiology, University of Sindh, Jamshoro 76080, Pakistan; qamar.abbas.qau@gmail.com; 3 Department of Pharmacognosy, College of Pharmacy, King Saud University, Riyadh 11451, Saudi Arabia; malajmii@ksu.edu.sa; 4 CEQUINOR (UNLP, CONICET-CCT La Plata), Departamento de Química, Facultad de Ciencias Exactas, Universidad Nacional de La Plata, Boulevard 120 e/60 y 64 N°1465, La Plata 1900, Argentina; erbenmf@gmail.com; 5 Department of Biological Sciences, College of Natural Sciences, Kongju National University, 56 Gongjudehak-Ro, Gongju, Chungnam 32588, Korea; mubashirhassan_gcul@yahoo.com (M.H.); hussain_solangi@yahoo.com (H.R.); dnalove@kongju.ac.kr (S.-Y.S.); 6 Pharmacognosy Group, Department of Medicinal Chemistry, Biomedical Center (BMC), Uppsala University, SE-751 23 Uppsala, Sweden; Hesham.El-Seedi@fkog.uu.se

**Keywords:** bis-azo Schiff bases, dual inhibitor, α-glucosidase inhibitor, α-amylase, antioxidant, SAR, chemo-informatics, kinetic mechanism, molecular docking

## Abstract

A series of symmetrical salicylaldehyde-bishydrazine azo molecules, **5a**–**5h**, have been synthesized, characterized by ^1^H-NMR and ^13^C-NMR, and evaluated for their in vitro α-glucosidase and α-amylase inhibitory activities. All the synthesized compounds efficiently inhibited both enzymes. Compound **5g** was the most potent derivative in the series, and powerfully inhibited both α-glucosidase and α-amylase. The IC_50_ of **5g** against α-glucosidase was 0.35917 ± 0.0189 µM (standard acarbose IC_50_ = 6.109 ± 0.329 µM), and the IC_50_ value of **5g** against α-amylase was 0.4379 ± 0.0423 µM (standard acarbose IC_50_ = 33.178 ± 2.392 µM). The Lineweaver-Burk plot indicated that compound **5g** is a competitive inhibitor of α-glucosidase. The binding interactions of the most active analogues were confirmed through molecular docking studies. Docking studies showed that **5g** interacts with the residues Trp690, Asp548, Arg425, and Glu426, which form hydrogen bonds to **5g** with distances of 2.05, 2.20, 2.10 and 2.18 Å, respectively. All compounds showed high mutagenic and tumorigenic behaviors, and only **5e** showed irritant properties. In addition, all the derivatives showed good antioxidant activities. The pharmacokinetic evaluation also revealed promising results

## 1. Introduction

**Figure 1:**
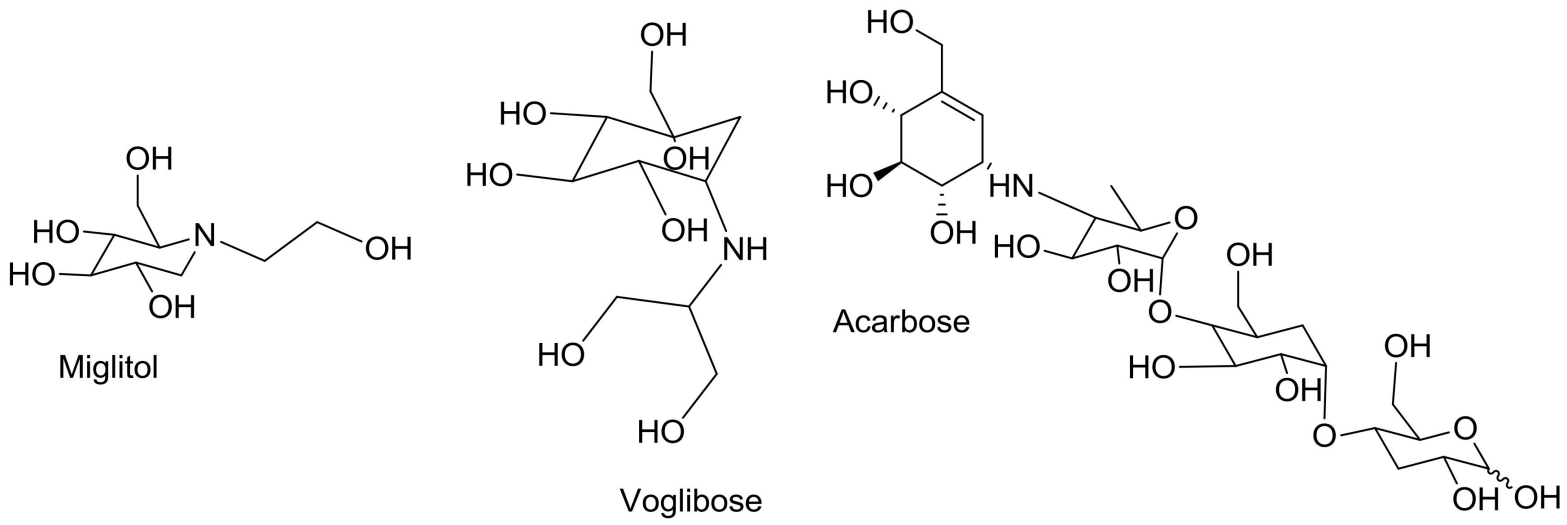
Structures of some of the available α-glucosidase inhibitors.

Diabetes mellitus (DM) is a metabolic disorder that poses a serious threat to human health across the globe [1]. In particular, type 2 diabetes mellitus (T2DM) is widely encountered in aged people [2]. An imbalance in the transport of glucose is the main factor leading to diabetes [3]. An abrupt increase in the glucose level is the major cause of T2DM. Changes in social norms and a lack of proper education regarding a balanced diet are among the main causes of DM [4]. According to the World Health Organization, at least 2.8% of the world’s population suffered from T2DM in 2000, and it is estimated that by 2030, this percentage will almost double [5]. The drugs approved by the U.S. FDA for the treatment of diabetes can be categorized into five classes: Biguanides, thiazolidinediones, sulfonylureas, meglitinides and α-glucosidase inhibitors (AGIs) [6]. The efficacy and sustainability of some drugs are not sufficient. Therefore, the identification of more effective and safer anti-diabetic drugs is of great importance. Several AIGs, such as acarbose (Glucobay^®^), voglibose (Volix^®^, Basen^®^) and miglitol (Glyset^®^) (Figure 1), can reversibly inhibit α-glucosidase, consequently delaying the absorption of sugars from the gut, and they have been used clinically for the treatment of DM [7].

There commercially available AGIs are associated with certain drawbacks, such as requiring tedious, multistep syntheses; for example voglibose is prepared via a 13-step procedure from (−) shikimic acid, and miglitol is prepared in 13 steps from (*R*)-methyl 2-benzamido-3-((*tert*-butyldimethylsilyl)oxy)propanoate. Moreover, these AGIs also show some serious adverse effects, including causing flatulence and diarrhoea [8].

To control postprandial glucose levels in T2DM patients, AGIs must be administered. AGIs assist in controlling the progress of hyperglycaemia and hyperinsulinaemia by diverting the workload from pancreatic cells. Postprandial hyperglycaemia (PPHG), if not cured properly and in a timely manner, can lead to life-threatening complications, such as cardiovascular disease and hypertension-related disorders [9]. Acarbose was the first AGI and was made commercially available in 1990. Japanese regulatory agencies approved voglibose as a new AGI in 1994, and two years later, American regulatory agencies approved miglitol as a potent anti-diabetic agent [10]. Among these three AGIs (acarbose, voglibose and miglitol), acarbose has promising effects on several intestinal enzymes; for example, it mainly inhibits α-amylases, including glucoamylase (90%), sucrose (65%), maltase (60%) and isomaltase (10%) [11]. α-Glucosidase is a membrane-bound enzyme found in intestinal cells that catalyses the production of glucose from a carbohydrate source [12] and α-Glucosidase cleaves α-glycosidic bonds. Glucosidase inhibitors can be used as first-line drugs for the treatment of T2DM, because they can lower the rate of carbohydrate absorption and suppress postprandial hyperglycaemia [13,14,15,16,17].

Schiff bases, also known as imines or azomethines, are considered a privileged class of organic compounds and have extensive applications in the fields of biology [18,19], catalysis [20], materials chemistry [21] and inorganic [22] and analytical chemistry [23]. These compounds possess a wide range of biological activities, such as antibacterial, antifungal, antimalarial, anticancer, antitubercular, anti-inflammatory, and antiviral activities [24]. Schiff bases are key precursors in the synthesis of a wide variety of metal complexes, and Schiff base-metal complexes are stable in nature and are useful in non-linear optics (NLOs). Salicylaldehyde-derived Schiff bases have attracted substantial attention, because they exhibit promising effects against microbes [25].

Azo dyes are key chromophores in the chemical industry as dyes and pigments [26], food additives [27], indicators [28], radical initiators [29] and therapeutic agents [30]. Azo azomethine compounds are commonly prepared by the condensation of a primary amine or hydrazine with an aromatic aldehyde or ketone-linked –N=N– chromophore [31]. Azo ligands are considered robust and chemically stable compounds, and they have been thoroughly investigated in the dye and photoelectrochemical fields [32]. Besides a number of negative features attached to azo compounds, such as toxicity and non-biodegradability, there are several favorable aspects that make them medically attractive compounds. These include the specific azoreduction in vivo, site specific drug delivery of polymeric azo compound in the colon diseases, such as colitis and irritable bowel syndrome Reductive degradation and subsequent splitting of the azo bond Occur in the colon, and therefore they are highly site-specific. Similarly, the azo pro-drugs are reduced to the corresponding amines that are exact therapeutics, for example, 5-aminosalicylic acid derivatives that exhibit anti-inflammatory and cytoprotective potency [33]. FP-21399 is another azo molecule which exhibits anti-HIV potency by inhibiting HIV envelope glycoprotein-mediated membrane fusion that precedes virus infiltration into the cell [34]. There are a limited number of studies on the anti-diabetic and pharmacodynamic properties of salicylaldehyde-derived bis-azo Schiff base ligands.

Herein, we develop a synthetic route to construct new bis-azo ligands using salicylaldehyde as a key precursor. The final products contain a bis-azo group, four aromatic rings, two rings bearing hydroxyl groups and two rings bearing electron-withdrawing substituents.

These ligands were selected for two reasons: (i) To provide new acyclic salicylaldehyde-based bis-azo-containing Schiff base ligands, and (ii) to investigate their inhibitory activities against α-amylase and α-glucosidase to uncover their antioxidant potential, the kinetics of their enzyme inhibition, their pharmacodynamics (chemo-informatics and Lipinski’s rule validation), their initial structure-activity relationships and their binding affinities by exploiting molecular docking.

## 2. Results and Discussion

**Scheme 1:**
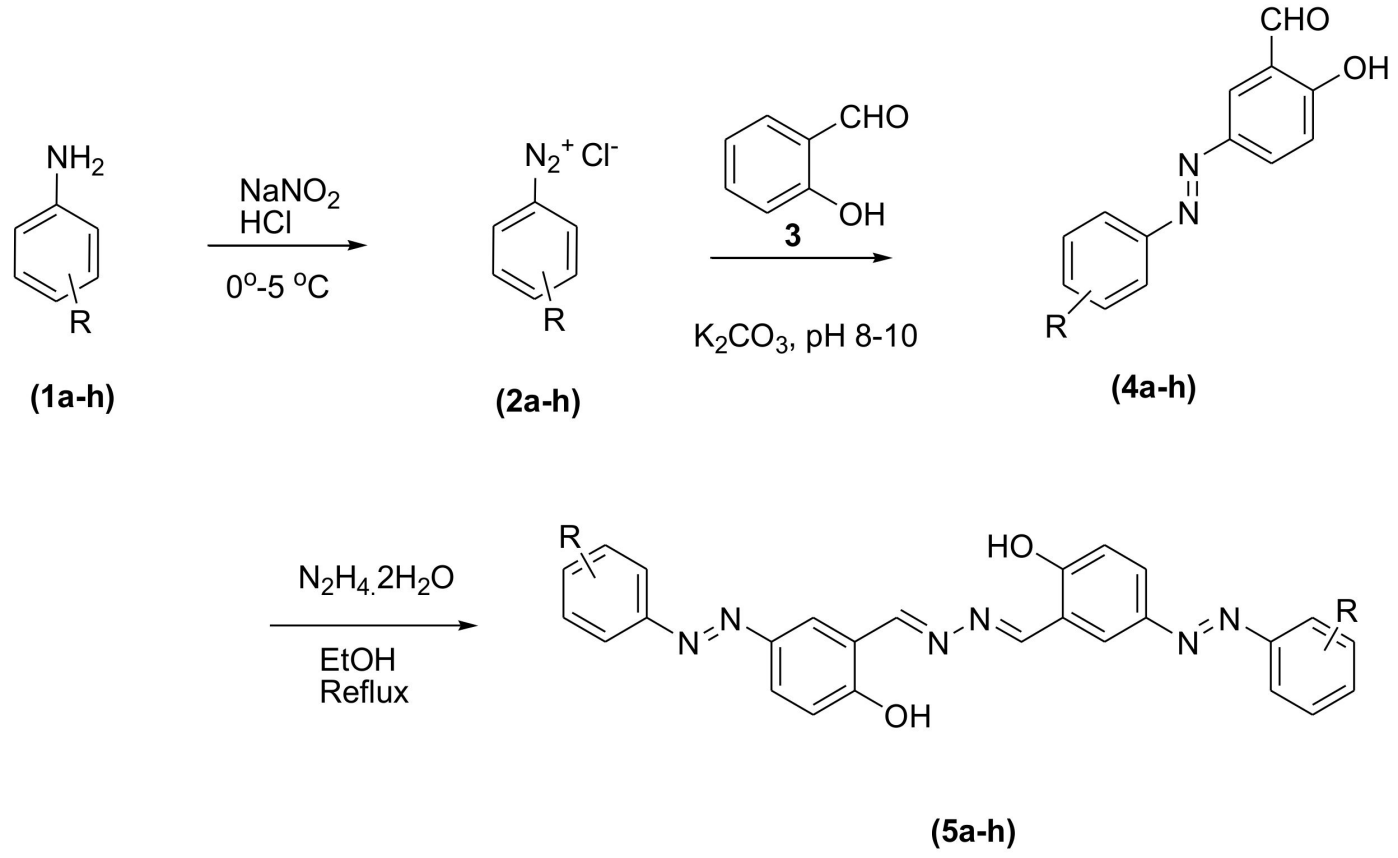
The synthetic route towards novel symmetrical compounds (**5a**–**h**).

**Scheme 2:**
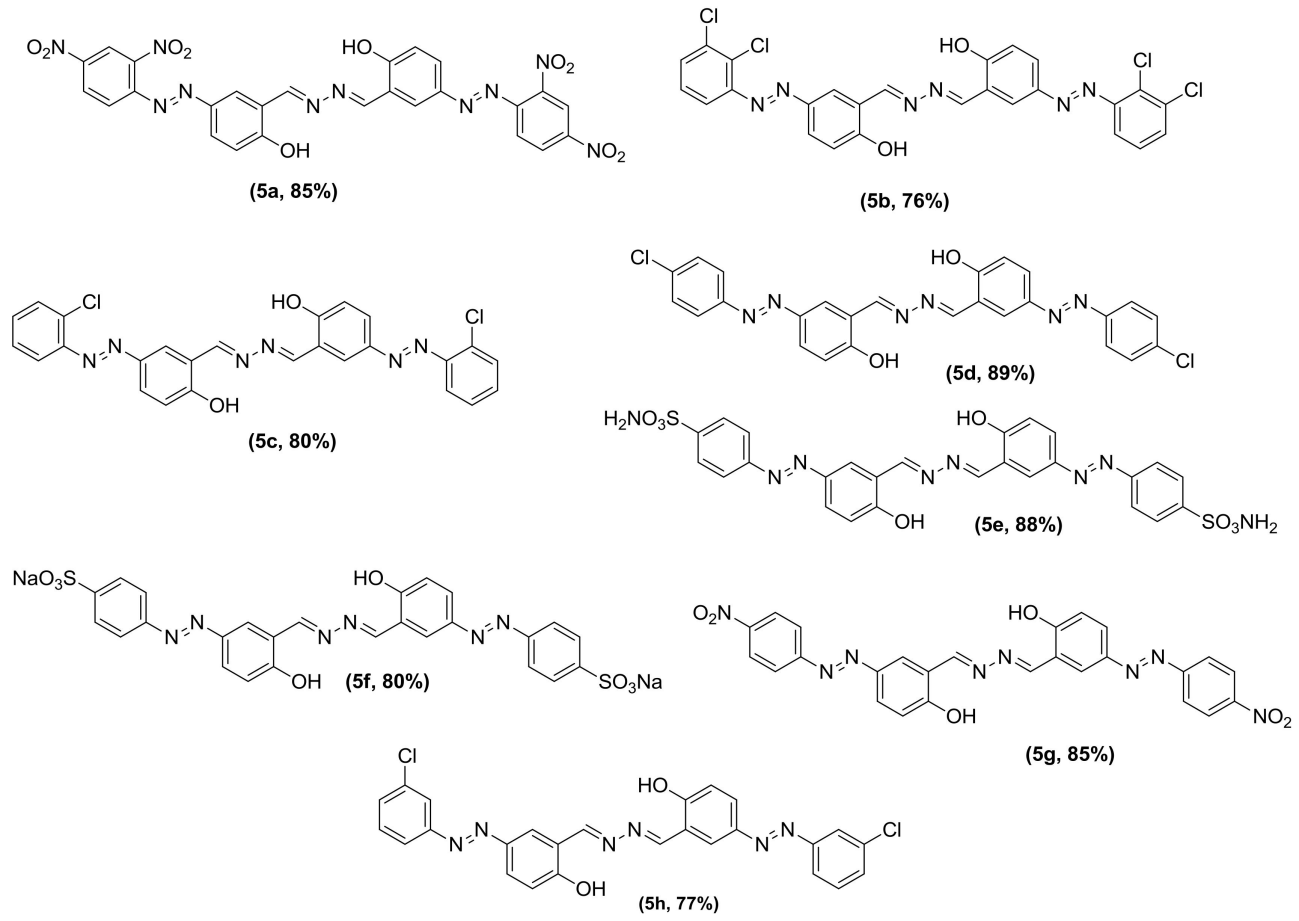
Structures and yields of synthesized symmetrical molecules (**5a**–**h**).

The synthesis of the bis-azo dyes containing azomethine is depicted in Scheme 1, and the structures and yields of the compounds are shown in Scheme 2. Appropriately substituted anilines (**1a**–**h**) were used as the starting materials, and the amine group was converted into the diazonium salt using NaNO_2_ and drops of HCl. The diazonium salts (**2a**–**h**) were then coupled with salicylaldehyde under mildly basic conditions to afford **4a**–**h**. The aldehyde groups of **4a**–**h** were converted to the corresponding imine with hydrazine hydrate using ethanol to afford the desired products (**5a**–**h**) in encouraging yields.

The synthesized compounds were characterized through ^1^H-NMR and ^13^C-NMR spectroscopy. The signal at approximately 9.56 ppm was attributed to the azomethine proton. The phenolic proton appeared at 5–6 ppm. The signals appearing at 7–8 ppm were attributed to protons on the phenyl ring. The signals in the ^13^C-NMR spectra from 160–170 ppm were assigned to the carbon of the imine moiety. The carbons bearing electron-withdrawing groups appeared downfield, and the sp^2^ carbons of the phenyl rings appeared from approximately 140–120 ppm.

Azomethine-linked salicylaldehyde is widely recognized as a bioactive moiety. These newly synthesized, bis-azo salicylaldehyde-based Schiff bases inhibit α-glucosidase and α-amylase to various degrees. To elucidate the electronic influence on enzyme inhibition, substrates representing almost all possible substitution patterns of the azo group on the phenyl ring were explored. The search for new, potent and safe anti-diabetic agents will remain an important and challenging task for medicinal chemists. Synthesized derivatives **5a**–**h** are structurally similar to each other, and they only differ in the position of the electron-withdrawing group on the phenyl ring. However, these ligands all share a common feature: The presence of two hydroxyl groups, which are pivotal to the inhibition of α-glucosidase and α-amylase. The azomethine moiety is considered one of the most intriguing structural entities in medicinal chemistry, because it can interact with the target protein of the enzyme via non-covalent interactions by serving as a hydrogen bond acceptor. Interestingly, compound **5g**, which is a non-halogenated derivative in this series, exhibited the highest inhibitory potential against both enzymes (α-glucoside and α-amylase), and it was a dual inhibitor of these two diabetes-promoting enzymes [35]. The IC_50_ values of **5g** were found to be several folds better than those of acarbose, which was used as a standard (Table 1).

Nitro-substitution is found in a number of marketed drugs such as Azomycin, Nifurtimox, Benznidazole, Tinidazole, Fexinidazole, Ventetoclax, Delamanid, Entacapone etc. Similarly, there are several therapeutic agents containing a nitro group with bioreductive potentials, such as Paclitaxel prodrug, Tarloxotinib, BTZ043, Evofosfamide, CB-1954 and KS119 [36]. Nevertheless, the nitro group is considered as both a pharmacophore and a toxicophore (hepatotoxic, mutagenic). Compound **5g** possessed a nitro group at the para position of the phenyl ring and showed the most potent inhibition. Compound **5d**, which possessed a chloride at the para position of the phenyl ring, showed the weakest inhibition in the case of α-glucosidase, indicating that the nature of the electronic group substantially influenced the inhibitory effect. A nitro group can induce great charge density by pulling electrons from the aromatic ring via a resonance effect, while the substituent on compound **5d** can also shift the electron cloud via an inductive effect, but the charge separation will be greater in the case of **5g**. In the case of α-amylase inhibition, compound **5e** displayed the weakest inhibition, which can be attributed to the presence of a sulfonamide group at the para position of the phenyl ring. All the compounds showed significant inhibition and can serve as lead molecules in the design of DM inhibitors.

### 2.1. Kinetic Analysis

**Figure 2:**
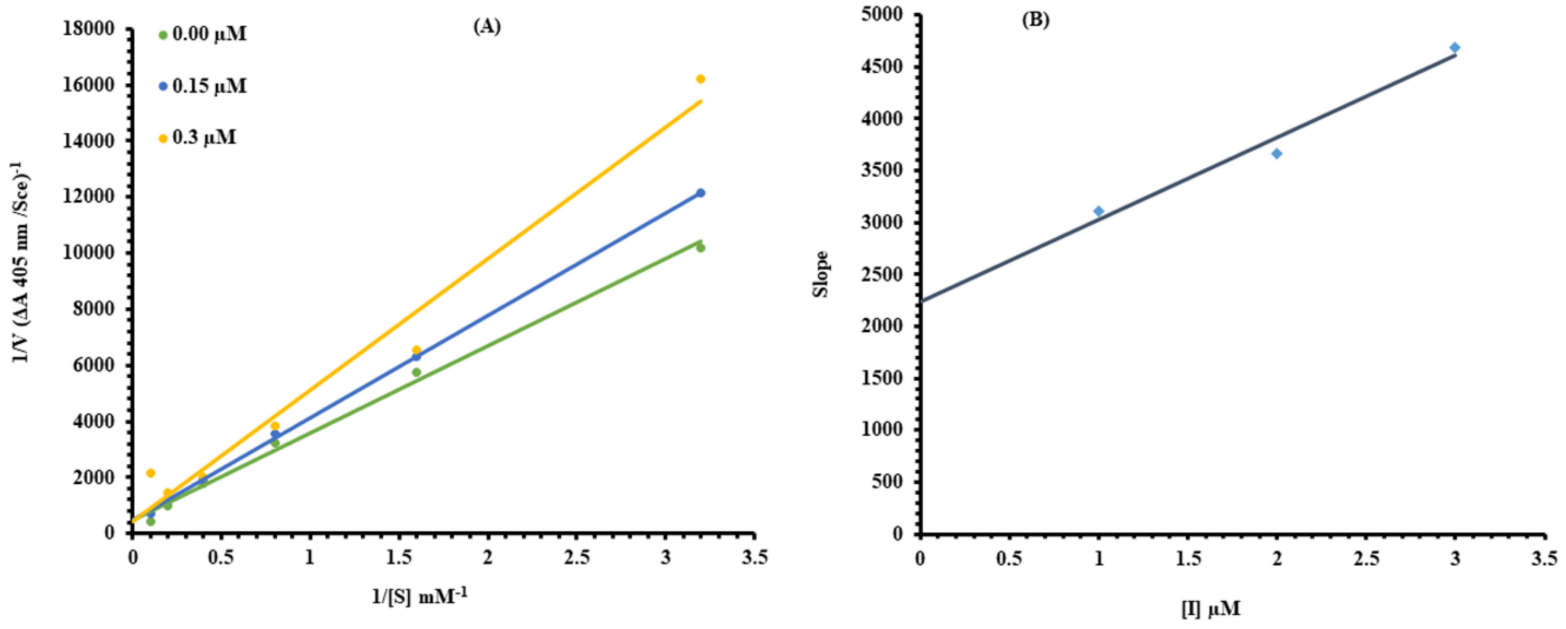
Lineweaver-Burk plots for the inhibition of α-glucosidase by compound 5g (**A**) The tested concentrations of 5g were 0.00, 0.15, and 0.3 µM; the concentrations of the substrate (p-nitrophenyl-α-D glucopyranoside) were 10, 5, 2.5, 1.25, 0.625, and 0.3125 mM. (**B**) The plots of the slope versus inhibitor concentration are shown as insets and were used to determine the inhibition constants.

To understand the inhibition mechanism of the synthesized compounds on α-glucosidase, an inhibition kinetics study was performed. The inhibition type and an inhibition constant of the most potent compound, **5g**, based on our IC_50_ results, were determined. In our evaluation of the kinetics of the enzyme, the Lineweaver-Burk plot of 1/V versus 1/[S] in the presence of different inhibitor concentrations gave a series of straight lines. The Lineweaver-Burk plot of compound **5g** showed that V_max_ remains the same without significantly affecting the slopes. K_m_ increases with increasing concentration, while V_max_ remains essentially constant. This behavior indicates that **5g** inhibits the α-glucosidase in a competitive manner (Figure 2A). Second, as shown in Figure 2B, the plot of the slope against the concentration of **5g** provided the EI dissociation constant. Ki was calculated from the concentration of **5g** versus the slope, and K_i_ was found to be 3.865 µM.

### 2.2. Free Radical Scavenging

**Figure 3:**
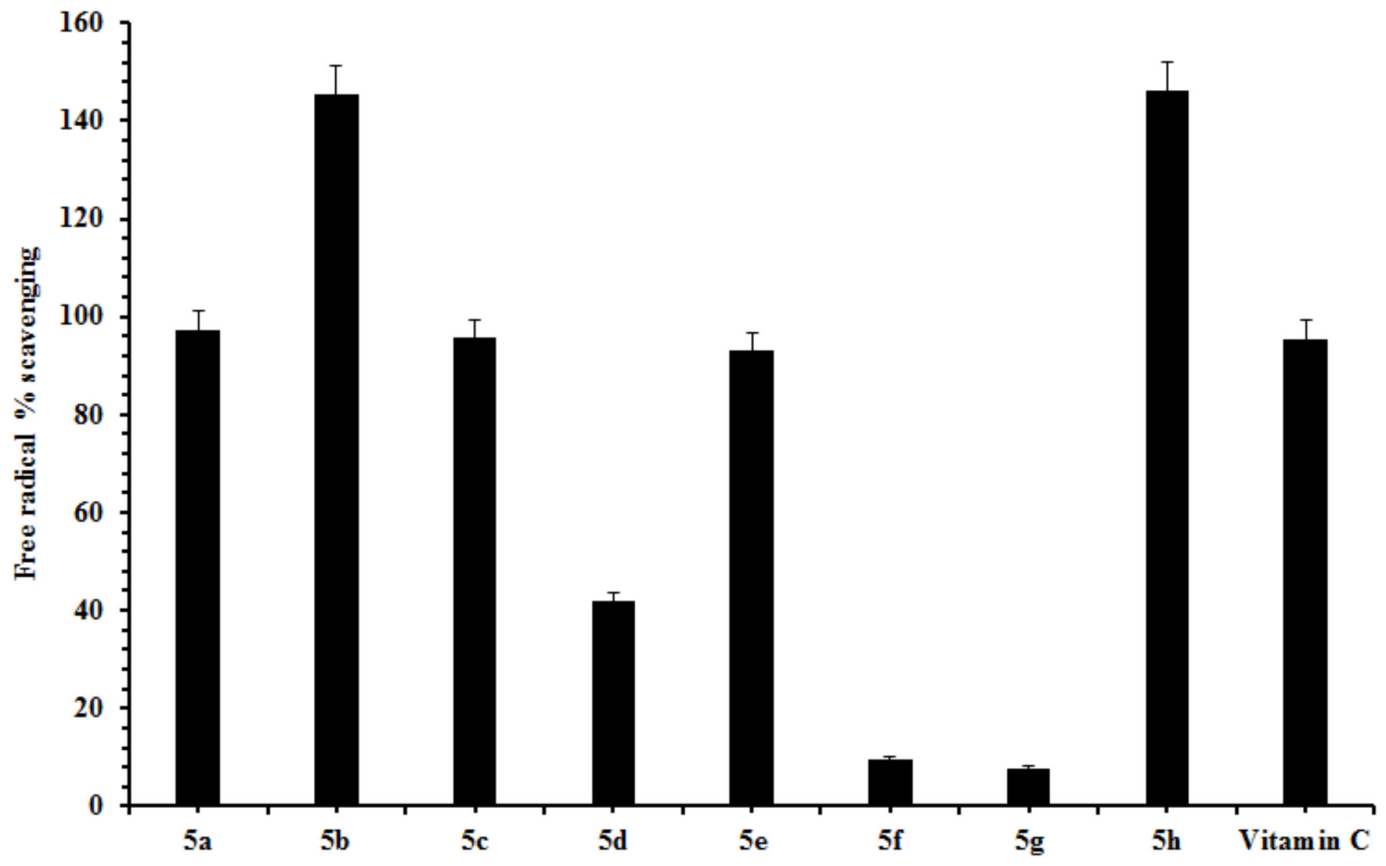
Free radical scavenging activity (%) of the synthesized compounds; values are presented as the mean ± SEM. All compound concentrations were 100 µg/mL.

The DPPH free radical scavenging ability of each of the synthesized compounds was evaluated. Compounds **5d**, **5f** and **5g** did not show significant scavenging potencies (%); however, the other compounds showed significant radical scavenging potentials at a concentration of 100 µg/mL (Figure 3).

### 2.3. Structural Assessment of α-Amylase and α-Glucosidase

**Figure 4:**
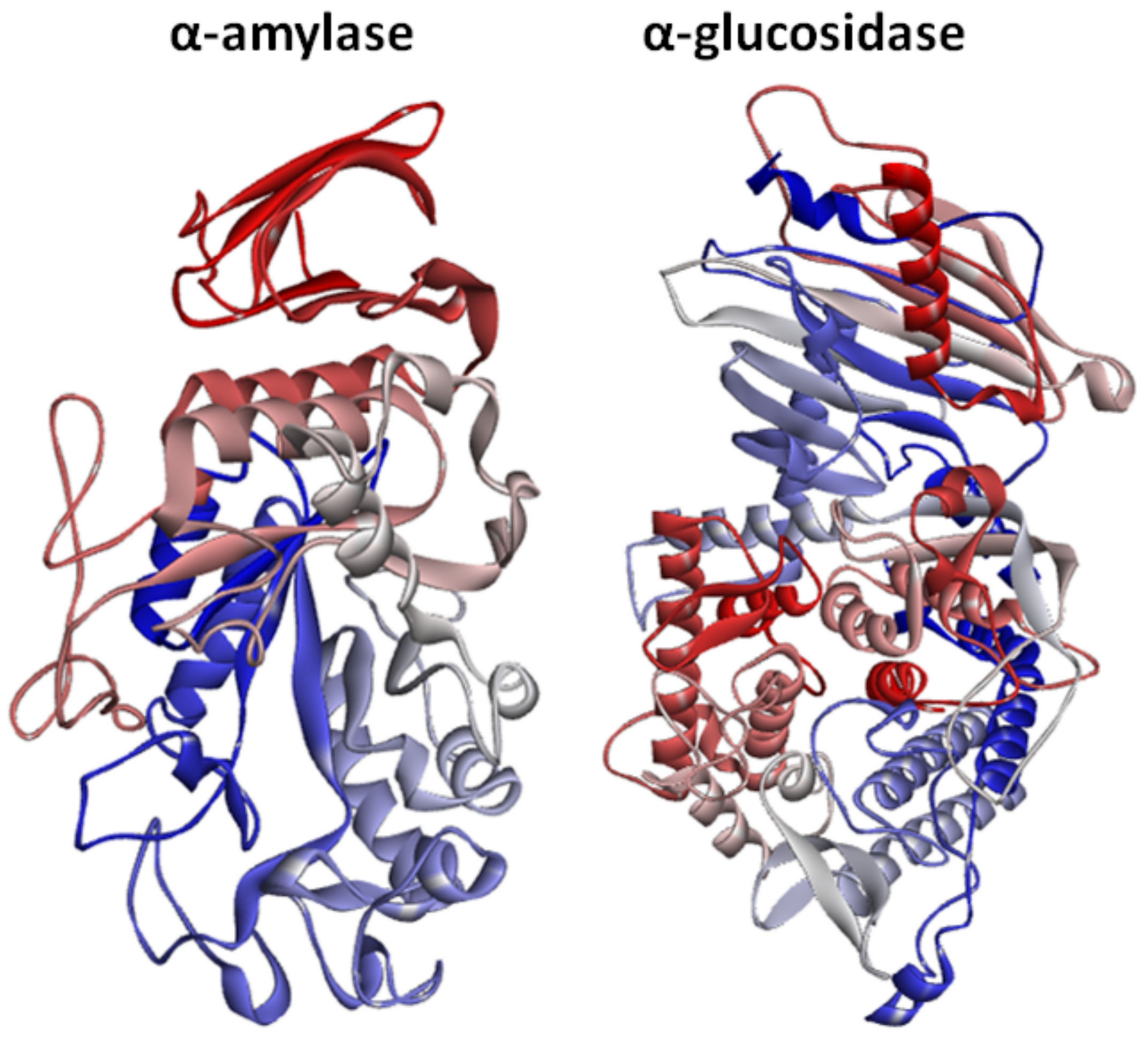
The crystal structures of α-amylase and α-glucosidase.

α-Amylase (EC#: 3.2.1.1) and α-glucosidase (EC#: 3.2.1.106) are a class of hydrolyzed proteins that are actively involved as receptor molecules and can be used as targets for therapeutics for the treatment of diabetes. Porcine pancreatic α-amylase contains 496 residues with 22% helices, 30% β sheets and 47% coil. α-Glucosidase comprises 811 amino acids with 35% helices, 25% β sheets and 38% coil. X-ray diffraction studies were used to confirm the structures of α-amylase and α-glucosidase to resolutions of 1.85 Å and 2.04 Å, respectively (Figure 4). The Ramachandran plots of α-amylase and α-glucosidase indicated that 97.1% and 97.6% of residues, respectively, were present in the well-resolved regions. The Ramachandran graphs showed the high accuracy of the phi (φ) and psi (ψ) angles among the coordinates of target proteins. The Ramachandran graphs of α-amylase and α-glucosidase are provided in Supplementary Figure S1A,B, respectively.

### 2.4. Chemo-Informatics and Lipinski’s Rule of Five (RO5) Assessments of the Ligands

The predicted properties, such as the molecular polar surface area (PSA), molar volume, density, polarizability, surface tension, molar refractivity and Lipinski rule violations, were evaluated to determine drug likeness Prior research has shown that PSA is a significant parameter for predicting drug absorption in drug discovery [37]. The molar refractivity and molecular lipophilicity of drug molecules are important for receptor binding, bioavailability and cellular uptake. Standard values for molar refractivity (40 to 130 cm^3^), molecular weight (160 to 480 g/mol) and PSA (<89 Å^2^) have been reported [38]. The predicted results showed that the molar refractivity and PSA values were comparable to the standard values as mentioned in Table 2. RO5 analysis was used to justify the therapeutic potential of the synthesized compounds. Hydrogen-bonding affinity is an important parameter for evaluating drug permeability [39], and the values of HBA > 10 and HBD > 5 for the ligands result in poor permeation in the body. Our chemo-informatics analyses showed that these ligands possess ≤ 10 HBA and < 5 HBD, which suggest good penetration within the body. Moreover, their log*P* values (−2.10) were also comparable to the standard values (< 5). However, there are many examples of RO5 violations among existing drugs [40,41]. The predicted properties are presented in Table 2.

### 2.5. Lead Optimization and Lipophilicity Values

To further evaluate the lead optimization potential of the synthesized compounds. The ligand efficiency (LE), lipophilic ligand efficiency (LLE) and lipophilicity-corrected ligand efficiency (LELP) values of all the synthesized compounds were predicted using the Data Warrior tool. Lipophilicity is a fundamental property for improving the efficacy of lead compounds and identifying drug candidates [42,43,44]. Hopkins studied the lipophilicities of different compounds and predicted standard values for LE, LLE and LELP on the basis of cLog*P* values [44]. Suggested acceptable standard values have been reported for LE (>~0.30 kcal/mole/HA), LLE (>~0.5 kcal/mol), and LELP (−10< to <10) [44,45]. The predicted LE values of the synthesized compounds were comparable to the standard values. Furthermore, all the compounds showed mutagenic and irritant effects. All the compounds showed potent mutagenic and tumorigenic behavior, whereas only **5e** showed irritant properties. The predicted values for all compounds along with their mutagenic and irritant effects are presented in Table 3.

### 2.6. Molecular Docking Analysis

**Figure 5:**
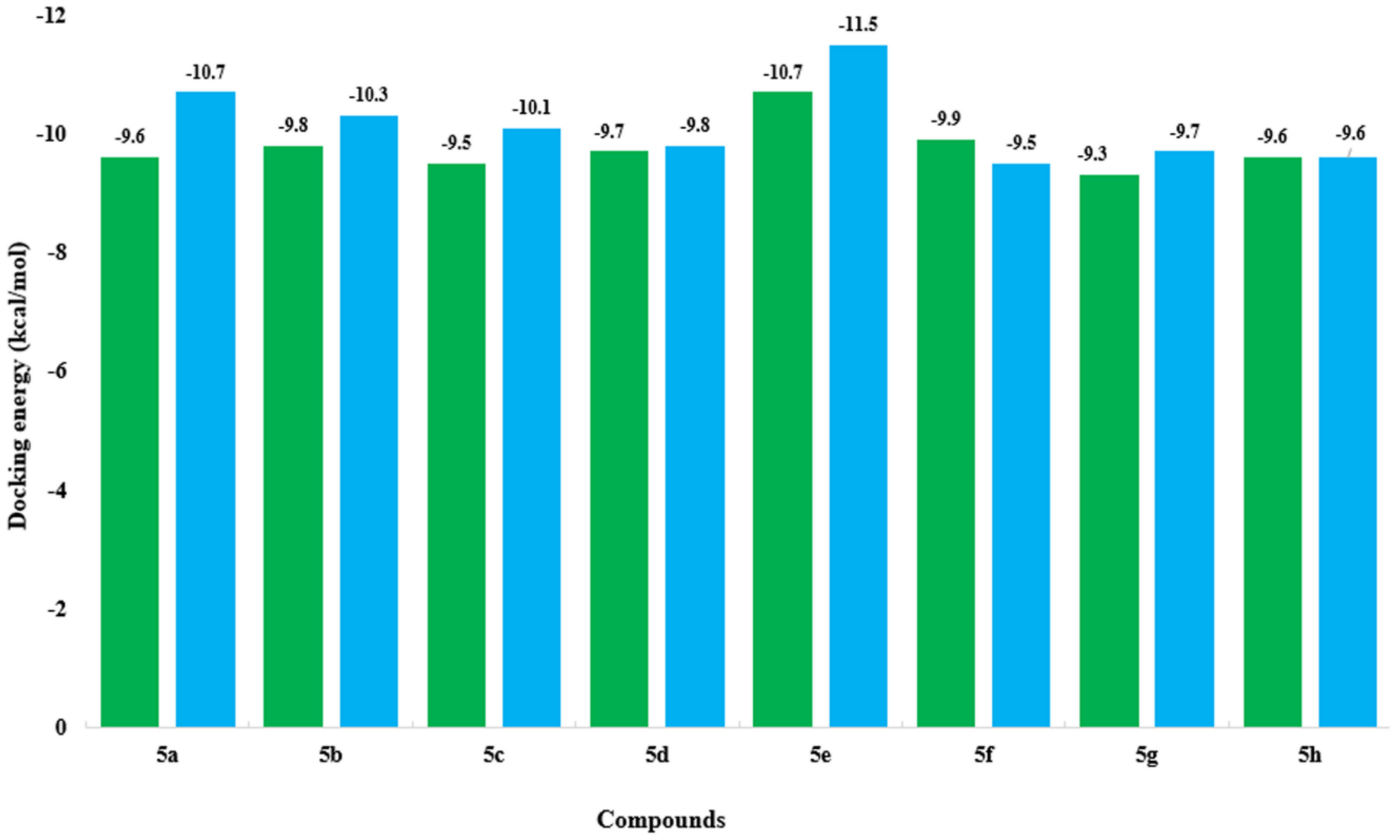
Energy graph of both α-amylase and α-glucosidase docking (the green bar shows α-amylase, and the blue bars show α-glucosidase).

The docked ligand-protein complexes were analysed based on their minimum energy values. The compounds showed good binding energy values against α-amylase and α-glucosidase (Figure 5). The energy values gave the binding potentials of synthesized compounds against the target proteins. The results showed that **5e** was the most active compound with the best binding energy values for both α-amylase and α-glucosidase (−10.70 and −11.50 kcal/mol, respectively) among the tested derivatives. Complexes with **5g** docked showed comparable docking energy values of −9.30 and −9.70 kcal/mol, respectively. The docking energy values of all the docked complexes were calculated using Equation (1), as mentioned in the Supplementary Materials. Prior research has shown that the standard error for Autodock is 2.5 kcal/mol. However, the predicted energy value for each of the docked complexes was less than the standard energy value. Although the synthesized compounds all had the same basic core, most ligands possess good energy values, and no large energy differences were observed. Compound **5g** was the most active in the in vitro analysis; therefore, a structure-activity relationship analysis was performed to evaluate its actual binding within the target proteins.

### 2.7. Structure-Activity Relationship (SAR) Analysis

**Figure 6:**
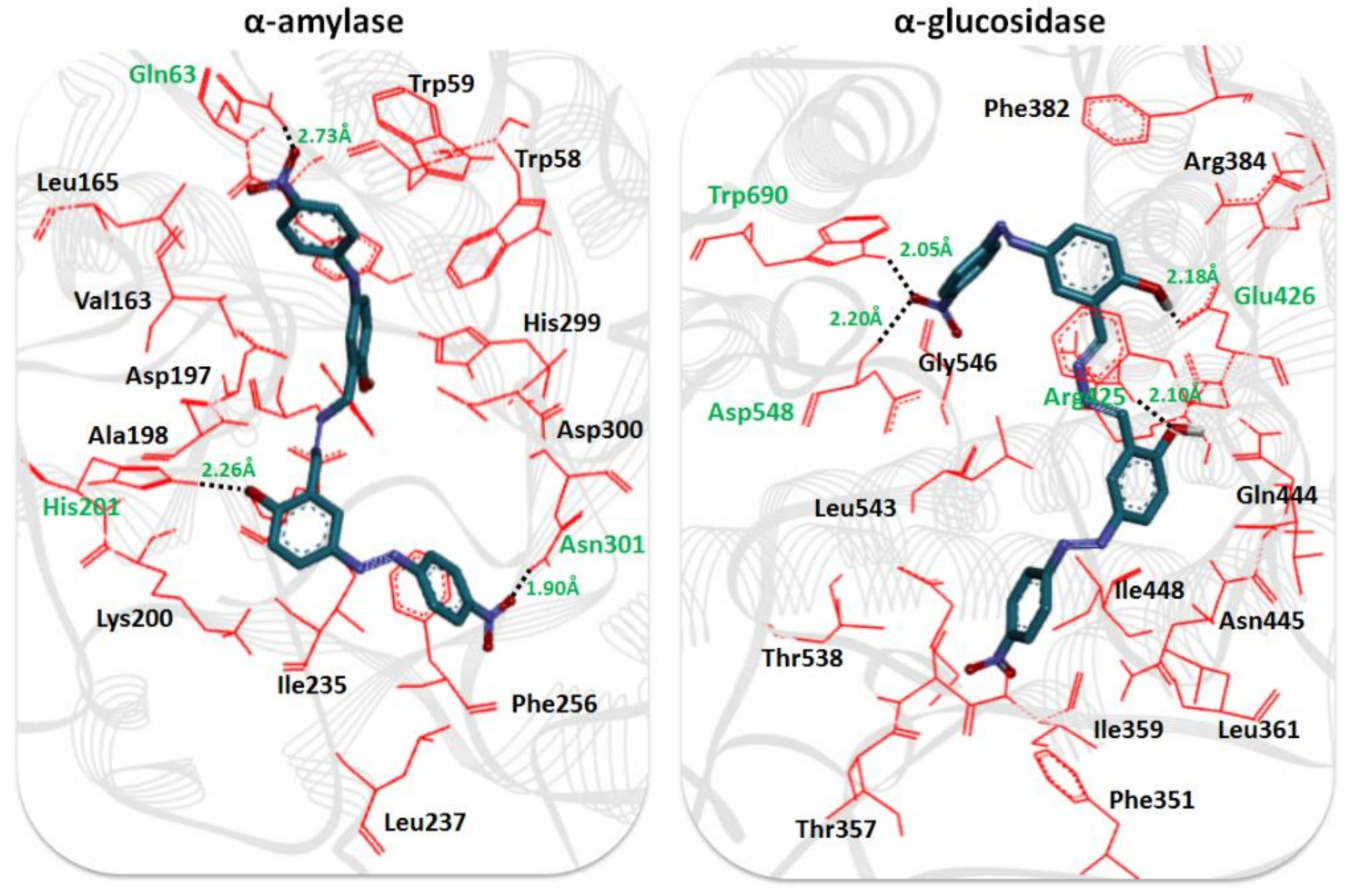
Docking of **5g** with α-amylase and α-glucosidase.

The active binding region in α-amylase has been reported previously [46]. The α-amylase docking results showed that **5g** binds within the active binding region of the target protein and forms three hydrogen bonds. The residues in the active site of α-amylase that actively participate in hydrogen bonding are Gln63, His201, and Asn301. The benzyl oxygen in **5g** forms a hydrogen bond with Gln63 with a bonding distance of 2.73 Å. Moreover, the hydroxyl and oxygen moieties on the benzene of **5g** form hydrogen bonds with His201 and Asn301 with bond distances of 2.26 and 1.90 Å, respectively. Our docking results were well correlated with a previously published article, confirming the accuracy of our docking results [47]. In α-glucosidase docking, Trp690, Asp548, Arg425, and Glu426 were the most active residues, as they formed hydrogen bonds to **5g** with distances of 2.05, 2.20, 2.10 and 2.18 Å, respectively (Figure 6). Based on our docking and kinetic results, it can be hypothesized that **5g** actively binds in the active region of the target protein and has great potential in the treatment of diabetes.

## 3. Experimental

### 3.1. Methods and Materials

The R_f_ values were determined using aluminium pre-coated silica gel plates (Kiesel 60 F254) from Merck (Darmstadt, Germany). Melting points were measured in open capillaries using a Stuart melting point apparatus (SMP3) and are uncorrected. ^1^H-NMR spectra were determined from DMSO or CDCl_3_ solutions at 300 MHz using a Bruker AM-300 spectrophotometer, and the ^13^C-NMR spectra were determined at 75 MHz using a Bruker 75 MHz NMR from DMSO-*d*_6_ or CDCl_3_ solutions. Elemental analyses were performed on a LECO CHNS-932 Elemental Analyzer (LECO Corporation, Saint Joseph, MI, USA). All the chemicals used for the syntheses of the compounds were commercially obtained and were used without additional purification.

### 3.2. General Procedure for the Synthesis of Phenolic Azo Dyes (***4a**–**4j***) and Their Condensation with Hydrazine Dihydrate (***5a**–**5j***)

Suitably substituted anilines (**1a**–**1h**) (0.01 mol) were dissolved in 20 mL of water and 3.5 mL of concentrated HCl with stirring at a temperature of 0–5 °C. A solution of NaNO_2_ (0.01 mL) in 10 mL of water was promptly added to the aniline solution with continuous and vigorous stirring. The reaction mixture was stirred for 1 h while maintaining the temperature in the same range. After 1 h, the reaction mixture was checked for the completeness with a paper chromatogram using water as the mobile phase. The dried chromatogram was sprayed with a solution of *p*-*N,N*-dimethyl aminobenzaldehyde in ethanol. Upon completion of the reaction, the diazonium salts (**2a**–**2h**) were stored in a freezer.

Salicylaldehyde **3** (0.01 mL) was dissolved in water (15 mL) with K_2_CO_3_ (1.5 g) and kept in an ice bath at 0–5 °C with stirring. The diazo solution was added dropwise to the stirred solution of salicylaldehyde over 45 min, and the pH was maintained above 8. The progress of the reaction was monitored by paper chromatography using H-acid solution in alkaline media. Upon completion, the solids were isolated by filtration and dried in an oven at 70 °C for 3 h to afford the 4-(benzeneazo)salicylaldehyde derivatives (**3a**–**3h**) in 85–92% yields. When mixtures of regioisomers were obtained, they were separated by column chromatography. Then, 0.01 mol (0.5 g) of hydrazine dihydrate (N_2_H_4_∙2H_2_O) was slowly added to a solution of 0.02 mol of the appropriate 4-(benzeneazo) salicylaldehyde derivative **(5a**–**5h)**. After refluxing the reaction mixture for 3 h, the mixture was cooled, and the precipitate was collected by filtration. The precipitate was washed several times with ethanol, crystallized from ethanol, and dried at 50 °C overnight (yields > 70%).

*(E)-2,2’-((1E,1’E)-Hydrazine-1,2-diylidenebis(methanylylidene))bis(4-((E)-(2,4-dinitrophenyl)diazenyl)phenol)* (**5a**)

Light yellow crystalline solid, mp = 275 °C, Yield 95%, R_f_ = 0.45 (*n*-Hexane: Ethyl acetate 6:4), ^1^H-NMR (DMSO-*d*_6_, 300 MHZ); δ (ppm) 11.81 (s, 2H, Ar-OH), 9.17 (s, 2H, =N-H), 8.69 (d, 2H, Ar-H, *J* = 1.9 Hz), 8.48 (d, 2H, Ar-H, *J* = 1.99 Hz), 8.01 (dd, 2H, Ar-H, *J* = 8.7 Hz, *J* = 2.1 Hz), 7.94 (dd, 2H, Ar-H, *J* = 8.6 Hz, *J* = 1.7 Hz), 7.87 (d, 2H, Ar-H, 8.9 Hz), 7.21 (d, 2H, Ar-H, *J* = 9 Hz), ^13^C-NMR (75 MHz DMSO-*d*_6_) δ (ppm), 161.94 (C=O), 161.83, 152.17, 150.62, 145.58, 144.87, 127.68, 127.21, 125.48, 122.35, 119.57, 118.27, 118.11,Anal. Calcd. for C_26_H_16_N_10_O_10_: C, 49.69; H, 2.57; N, 22.29; found: C, 49.60; H 2.60; N, 22.25.

*(E)-2,2’-((1E,1’E)-Hydrazine-1,2-diylidenebis(methanylylidene))bis(4-((E)-(2,3-dichlorophenyl)diazenyl)phenol)* (**5b**)

Dark yellow crystalline solid, mp= 222 °C, Yield 89%, R_f_ = 0.62 (*n*-Hexane: Ethyl acetate 6:4), ^1^H-NMR (CDCl_3_, 300 MH_Z_); δ (ppm) 11.82 (s, 2H, Ar-OH), 9.15 (s, 2H, =N-H), 8.40 (d, 2H, Ar-H, *J* = 2.0 Hz), 7.96 (dd, 2H, Ar-H, *J* = 8.7 Hz, *J* = 2.1 Hz), 7.89–7.59 (m, 6H, Ar-H), 7.21 (d, 2H, Ar-H, *J* = 8.92 Hz), ^13^C-NMR (75 MHz CDCl_3_) δ (ppm), 161.96 (C=O), 161.84, 154.19, 150.61, 130.11, 133.21, 128.64, 127.21, 125.48, 122.36, 119.53, 118.11, 116.21 Anal. Calcd. for C_26_H_16_Cl_4_N_6_O_2_: C, 53.27; H, 2.75; N, 14.34; found: C, 53.20; H, 2.83; N, 14.40.

*(E)-2,2’-((1E,1’E)-Hydrazine-1,2-diylidenebis(methanylylidene))bis(4-((E)-(2-chlorophenyl)diazenyl)phenol)* (**5c**)

Dark reddish-orange crystalline solid, mp = 195 °C, Yield 85%, R_f_ = 0.75 (*n*-Hexane: Ethyl acetate 6:4), ^1^H-NMR (CDCl_3_, 300 MH_Z_); δ (ppm) 11.81 (s, 2H, Ar-OH), 9.14 (s, 2H, =N-H), 8.38 (d, 2H, Ar-H, *J* = 2.0 Hz), 7.92 (dd, 2H, Ar-H, *J* = 8.60 Hz, *J* = 2.0 Hz), 7.89–7.49 (m, 8H, Ar-H), 7.20 (d, 2H, Ar-H, *J* = 8.98 Hz), ^13^C-NMR (75 MHz CDCl_3_) δ (ppm) 161.90 (C=O), 161.82, 152.12, 150.51, 127.66, 127.21, 125.47, 132.22, 124.22, 129.26,128.38, 119.58, 118.11Anal. Calcd. for C_26_H_18_C_l2_N_6_O_2_: C, 60.36; H, 3.51; N, 16.24; found: C, 60.42; H, 3.59; N, 16.31.

*(E)-2,2’-((1E,1’E)-Hydrazine-1,2-diylidenebis(methanylylidene))bis(4-((E)-(4-chlorophenyl)diazenyl)phenol)* (**5d**)

Light yellow crystalline solid, mp = 205 °C, Yield 86%, R_f_ =0.70 (*n*-Hexane: Ethyl acetate 6:4); ^1^H-NMR (CDCl_3_, 300 MH_Z_); δ (ppm) 11.81 (s, 2H, Ar-OH), 9.14 (s, 2H, =N-H), 8.37 (d, 2H, Ar-H, *J* = 2.1 Hz), 7.92 (dd, 2H, Ar-H, *J* = 8.61 Hz, *J* = 2.1 Hz), 7.79 (dd, 8H, Ar-H, *J* = 8.51 Hz), 7.19 (d, 2H, Ar-H, *J* = 8.98 Hz), ^13^C-NMR (75 MHz CDCl_3_) δ (ppm) 162.89 (C=O), 161.83, 152.11, 150.59, 136.21, 124.11, 129.21, 127.19, 125.46, 119.57, 118.10, Anal. Calcd. for C_26_H_18_C_l2_N_6_O_2_: C, 60.36; H, 3.51; N, 16.24; found: C, 60.44; H, 3.60; N, 16.29.

*4,4’-((1E,1’E)-(((1E,1’E)-Hydrazine-1,2-diylidenebis(methanylylidene))bis(4-hydroxy-3,1-phenylene))bis(diazene-2,1-diyl))dibenzenesulfonamide* (**5e**)

Dark orange crystalline solid, mp = 225 °C, Yield 76%, R_f_ =0.70 (Chloroform: Ethanol 9:1); ^1^H-NMR (DMSO-*d*_6_, 300 MH_Z_); δ (ppm) 11.83 (s, 2H, Ar-OH), 9.12 (s, 2H, =N-H), 8.38 (d, 2H, Ar-H, *J* = 2.0 Hz), 7.99 (dd, 2H, Ar-H, *J* = 8.6 Hz, *J* = 2.0 Hz), 7.81 (m, 8H, Ar-H), 7.55 (s, 4H), 7.21 (d, 2H, Ar-H, *J* = 8.94 Hz), ^13^C-NMR (75 MHz DMSO-*d*_6_) δ (ppm) 161.93 (C=O), 161.82, 152.16, 150.59, 145.61, 127.65, 127.19, 125.46, 122.35, 119.58, 118.09, Anal. Calcd. for C_26_H_22_N_8_O_6_S_2_: C, 51.48; H, 3.66; N, 18.47; S, 10.57; found: C, 51.42; H, 3.64; N, 18.43; S, 10.52.

*Sodium 4,4’-((1E,1’E)-(((1E,1’E)-hydrazine-1,2-diylidenebis(methanylylidene))bis(4-hydroxy-3,1-phenylene))bis(diazene-2,1-diyl))dibenzenesulfonate* (**5f**)

Bright yellow crystalline solid, m.p = 240 °C, Yield 80 %, R_f_ = 0.55 (Chloroform: Ethanol 9:1), ^1^H-NMR (DMSO-*d*_6_, 300 MH_Z_); δ (ppm) 11.84 (s, 2H, Ar-OH), 9.16 (s, 2H, =N-H), 8.40 (d, 2H, Ar-H, *J* = 2.1 Hz), 7.99 (dd, 2H, Ar-H, *J* = 8.7 Hz, *J* = 2.1 Hz), 7.82 (m, 8H, Ar-H), 7.23 (d, 2H, Ar-H, *J* = 9 Hz), ^13^C-NMR (75 MHz DMSO-*d*_6_) δ (ppm) 161.96 (C=O), 161.84, 152.17, 150.61, 145.59, 127.67, 127.20, 125.48, 122.36, 119.59, 118.11, Anal. Calcd. forC_26_H_18_N_6_Na_2_O_8_S_2_: C, 47.85; H, 2.78; N, 12.88; S, 9.83; Found: C, 47.81; H, 2.72; N, 12.83; S, 9.78.

*(E)-2,2’-((1E,1’E)-Hydrazine-1,2-diylidenebis(methanylylidene))bis(4-((E)-(4-nitrophenyl)diazenyl)phenol)* (**5g**)

Dark brown crystalline solid, m.p =215 °C, Yield 85%, R_f_ =0.45 (*n*-Hexane: Ethyl acetate 6:4), ^1^H-NMR (DMSO-*d*_6_, 300 MHZ); δ (ppm) 11.82 (s, 2H, Ar-OH), 9.14 (s, 2H, =N-H), 8.40 (d, 2H, Ar-H, *J* = 1.9 Hz), 7.98 (dd, 2H, Ar-H, *J* = 8.7 Hz, *J* = 2.1 Hz), 7.98 (dd, 8H, Ar-H), 7.21 (d, 2H, Ar-H, *J* = 8.9 Hz), ^13^C-NMR (75 MHz DMSO-*d*_6_) δ (ppm) 161.95 (C=O), 161.82, 152.14, 150.59, 145.57, 127.67, 127.19, 125.46, 122.35, 119.56, 118.09, Anal. Calcd. for C_26_H_18_N_8_O_6_: C, 57.99; H, 3.37; N, 20.81; found: C, 57.91; H, 3.29; N, 20.89.

*(E)-2,2’-((1E,1’E)-Hydrazine-1,2-diylidenebis(methanylylidene))bis(4-((E)-(3-chlorophenyl)diazenyl)phenol)* (**5h**)

Dark yellow crystalline solid, m.p = 210 °C, Yield 87%, R_f_ = 0.65 (*n*-Hexane: Ethyl acetate 6:4), ^1^H-NMR (CDCl_3_, 300 MHZ); δ (ppm) 11.80 (s, 2H, Ar-OH), 9.08 (s, 2H, =N-H), 8.39 (d, 2H, Ar-H, *J* = 1.9.0 Hz), 7.98 (dd, 2H, Ar-H, *J* = 8.59 Hz, *J* = 2.1 Hz), 8.12-7.76 (dd, 8H, Ar-H, *J* = 8.61 Hz), 7.21 (d, 2H, Ar-H, *J* = 8.90 Hz), ^13^C-NMR (75 MHz CDCl_3_) δ (ppm) 161.96 (C=O), 161.84, 152.18, 150.61, 133.09, 130.89, 130.24, 127.20, 125.48, 123.44, 121.02, 119.59, 118.11, Anal. Calcd. for C_26_H_18_C_l2_N_6_O_2_: C, 60.36; H, 3.51; N, 16.24; found: C, 60.44; H, 3.59; N, 16.32.

### 3.3. Biological Activity Methods

#### 3.3.1. α-Glucosidase Inhibition Assay

The α-glucosidase inhibitory activities of the synthesized compounds were evaluated following the method described by Saleem et al., 2014 [33]. Briefly, solutions of the substrate (5 mM, *p*-nitrophenyl-α-d-glucopyranoside, Sigma, St. Louis, MO, USA) and α-glucosidase (Sigma, St. Louis, MO, USA) were prepared in 100 mM phosphate buffer (pH 7.0) containing 0.2 g/L NaN_3_ and 2 g/L bovine serum albumin. The assay mixtures contained the compound (inhibitors), substrate and enzyme solutions. First, 10 µL of the test compound (dissolved in 1% DMSO) and 50 µL of the enzyme solution were added to a 96-well microplate, and the assay plate was incubated for 5 min at room temperature. Then, 50 µL of the substrate solution was added, and the mixtures were incubated for 10 min at 37 °C. Finally, the reactions were terminated by adding 100 µL of sodium carbonate solution (100 mM). The blank was prepared by adding all the components except the enzyme. The absorbance of was reaction mixture was recorded at 405 nm using a microplate reader (OPTI_Max_, Tunable Micro Plate Reader, Sunnyvale, CA, USA). Acarbose was used as a reference drug. All the reactions were performed in triplicate and repeated three times. Percentage inhibition was calculated using the following equation,

Inhibition (%) = [(B − S)/B] × 100(1)

Here, B and S are the absorbances of the blank and sample, respectively. The IC_50_ values were calculated by the program Prism 5.0 (GraphPad, San Diego, CA, USA).

#### 3.3.2. α-Amylase Inhibition Assay

The α-amylase inhibitory activity of sinigrin was determined according to a previously reported method [33] with slight modifications. Briefly, 40 μL of a solution of the test inhibitor and 40 μL of enzyme solution (α-amylase from porcine pancreas (Sigma A3176-500KU, 089K1661) in a buffer of 0.02 M sodium phosphate at pH 6.9 with 0.006 M sodium chloride) were added to a 1.5-mL Eppendorf tube and incubated for 10 min at room temperature. Then, 40 μL of a starch solution (1% in DMSO) was added to the pre-incubated tubes, and the solutions were incubated for an additional 10 min at 25 °C. Then, 100 μL of DNSA colouring reagent (10 g of sodium potassium tartrate, 1 g of 3,5-dinitrosalicylic acid and 20 mL of 2 N NaOH to a final volume of 100 mL in distilled water) was added, and the mixture was incubated in a boiling water bath for 5 min. After cooling to room temperature, the reaction mixture was diluted to 500 μL using distilled water, and the absorbance was recorded at 540 nm. For non-enzymatic reactions, the assays were performed with a blank containing all of the components except the enzyme. The values of the % inhibition and IC_50_ were calculated from the results of the assays using the same procedure as that described for the α-glucosidase inhibition assays.

#### 3.3.3. Free radical Scavenging Assay

Radical scavenging activity was determined by modifying a previously reported 2,2-diphenyl-1-picrylhydrazyl (DPPH) assay [34,35]. The assay solution consisted of 100 µL of DPPH solution (150 µM), 20 µL of the test compounds solution at increasing concentrations, and enough DMSO to bring the volume of solution in each well to 200 µL. The reaction mixture was then incubated for 30 min at room temperature. Ascorbic acid (vitamin C) was used as a reference inhibitor. The assay measurements were carried out using a micro plate reader (OPTI_Max_, Tunable) at 520 nm. The reaction rates were compared, and the percentages of inhibition caused by the presence of the test inhibitors were calculated. Each concentration was analysed in three independent experiments run in triplicate.

#### 3.3.4. Kinetic Study of α-Glucosidase

Based on the IC_50_ values, the most potent inhibitor, **5g**, was selected for kinetic analysis. A series of experiments was performed to calculate the inhibition kinetics of **5g**. The tested concentrations of **5g** were 0.00, 0.15 and 0.3. The concentration of substrate, *p*-nitrophenyl-α-d-glucopyranoside, was between 0.3125 and 10 mM in all the kinetics experiments. The pre-incubation and measurement times were the same as those discussed in the α-glucosidase inhibition assay procedure. The maximal initial velocity was determined from the initial linear portion of absorbance in the first five minutes following the addition of the enzyme at 30 s intervals. The type of inhibition of the enzyme was assessed by preparing Lineweaver-Burk plots of the inverse of the velocities (1/V) versus the inverse of the substrate concentration 1/[S] mM^−1^. The EI dissociation constant, Ki, was determined by the secondary plot of 1/V versus the inhibitor concentration.

### 3.4. Computational Methodology

#### 3.4.1. Selection of Target Proteins from the PDB

The three-dimensional (3D) structures of α-amylase and α-glucosidase, with PDBIDs of 1DHK and 4J5T, respectively, were retrieved from the Protein Data Bank (PDB) (www.rcsb.org). The energies of the target proteins were minimized with an Amber force field by using a conjugate gradient algorithm in UCSF Chimera 1.10.1 (Pettersen et al., 2006). The overall protein architecture and statistical percentages of helices, β-sheets, coils and turns were retrieved from the online server VADAR 1.8 (Willard et al., 2003). The Discovery Studio 4.1 Client (D. Studio, 2008) was used as a visualizing tool to generate graphical depictions of the target proteins.

#### 3.4.2. In-Silico Design of Ligand Structures

The synthesized ligands were sketched using the ACD/ChemSketch tool and minimized using UCSF Chimera 1.10.1. The basic biological properties, such as molecular weight (g/mol), numbers of hydrogen bond acceptors and donors (HBA/D), Log*P*, number of stereo centres, molecular volume (A^3^), molar refractivity density, polarizability and drug likeness score were evaluated using the online computational tools Molsoft (http://www.molsoft.com/) and Molinspiration (http://www.molinspiration.com/). Moreover, Lipinski’s rule of five was also considered to evaluate the efficacy of the ligand structures. Furthermore, the ligand efficiency (LE), lipophilic ligand efficiency (LLE) and lipophilicity-corrected ligand efficiency (LELP) values of all the ligands were calculated using the Data Warrior tool. Moreover, the mutagenic and irritant risks were assessed using the Data Warrior tool.

#### 3.4.3. Ligand-Based Docking Simulation

Molecular docking experiments were conducted on all the synthesized ligands against α-amylase and α-glucosidase using the PyRx docking tool (Dallakyan and Olson, 2015). The grid box-centred parametric values for α-amylase were adjusted as x = −18.44, y = −20.91, z = 8.22, while the size values for all coordinates were adjusted as x = 77.93, y = 68.98, and z = 103.65, respectively. Similarly, for α-glucosidase, the grid values were adjusted as center_x = 102.22, center_y = 40.10, center_z = 18.02, while the size values were adjusted as size_x = 57.60, size_y = 59.83 and size_z = 72.29, respectively. A default exhaustiveness value = 8 was used in both docking studies to maximize the binding conformational analysis. The docking poses (100 numbers of runs) for each docking study were adjusted to obtain the best docking results. The selected compound was docked separately and evaluated based on the lowest binding energy (Kcal/mol) and the structure-activity relationship. Graphical depictions of all the docking complexes were prepared using Discovery Studio (2.1.0).

## 4. Conclusions

A series of new bis-azo dyes based on Schiff bases (**5a**–**5h**) were synthesized and characterized through elemental and spectroscopic techniques. The abilities of the synthesized compounds to inhibit α-glucosidase and α-amylase were evaluated. Compounds **5a**–**5h** all significantly inhibited both of these enzymes. Derivatives **5a**–**5h** also showed good antioxidant activities. Kinetic studies were performed to elucidate the modes of inhibition of α-amylase and α-glucosidase, and the most potent derivative, **5g**, was a dual inhibitor of these enzymes via competitive inhibition. The binding modes of ligands with the target proteins were explored by molecular docking studies. Pharmacokinetics and chemo-informatics evaluations revealed that the properties of the molecules were similar, and a few of the derivatives in this series can serve as lead molecules in the design of drugs for the treatment of DM.

## Figures and Tables

**Table 1:** α-Glucosidase and α-amylase inhibitory activities of compounds **5a**–**h**.

Inhibitor	α-Glucosidase (µM)	α-Amylase (µM)
**5a**	0.547 ± 0.0289	8.541 ± 0.653
**5b**	5.345 ± 0.2826	28.373 ± 2.171
**5c**	8.061 ± 0.4263	9.5482 ± 0.730
**5d**	19.521 ± 1.0321	9.7183 ± 0.863
**5e**	0.367 ± 0.01941	28.4828 ± 2.081
**5f**	1.841 ± 0.09738	4.2861 ± 0.328
**5g**	0.359 ± 0.0189	0.4379 ± 0.042
**5h**	0.400 ± 0.0211	0.5902 ± 0.012
**Acarbose**	6.109 ± 0.329	33.178 ± 2.392

Values are expressed as the mean ± SEM; SEM = Standard Error of the Mean.

**Table 2:** Cheminformatics Properties.

Properties	5a	5b	5c	5d	5e	5f	5g	5h
Mol. Weight (g/mol)	632.14	584.01	516.09	516.09	638.10	652.04	540.15	516.09
No. HBA	16	8	8	8	16	14	12	8
No. HBD	6	2	2	2	6	2	4	2
Mol. Log*P*	6.77	11.01	9.82	10.06	4.42	6.26	7.82	10.06
No of SC	0	0	0	0	0	0	0	0
Mol. Vol (A^3^)	536.68	477.50	446.16	448.00	510.16	515.19	474.01	448.15
Molar Refractivity	154.84	150.61	141.40	141.40	158.32	N/A	143.52	141.40
Density	1.65	1.47	1.36	1.36	1.57	N/A	1.45	1.36
Polarizability	61.38	59.70	56.05	56.05	62.76	N/A	56.89	56.05
Drug Likeness Score	−1.49	−0.51	−0.67	−0.54	−1.22	−1.33	−1.16	−0.73

Abbreviation: Log*P* = Lipophilicity of partition coefficient, SC = stereo centers.

**Table 3:** Predicted ligand efficacy values.

Ligands	cLog*P*	LE	LLE	LELP	Mutagenic	Tumorigenic	Irritant
**5a**	4.8122	0.24806	3.5055	19.399	high	high	none
**5b**	10.923	0.28743	−2.960	38.001	high	high	none
**5c**	9.7106	0.30535	−1.697	31.802	high	high	none
**5d**	9.7106	0.30535	−1.697	31.802	high	high	none
**5e**	7.027	0.25421	1.1262	27.642	high	high	high
**5f**	1.5448	0.28781	7.2633	5.3675	high	high	none
**5g**	6.6554	0.28044	1.5214	23.732	high	high	none
**5h**	9.7106	0.30535	−1.697	31.802	high	high	None

## Supplementary Materials

Supplementary Materials are available online.
