# Supplementary material for: Novel C-2 Symmetric Molecules as α-Glucosidase and α-Amylase Inhibitors: Design, Synthesis, Kinetic Evaluation, Molecular Docking and Pharmacokinetics
Source: Molecules. 2019 Apr 17;24(8):1511. doi: 10.3390/molecules24081511 (PMC6515238; doi:10.3390/molecules24081511)
Supplement: Supplementary file 1 [file molecules-24-01511-s001.pdf]

Novel C-2 Symmetric Molecules as α-Glucosidase and α-Amylase Inhibitors: Design, Synthesis, Kinetic Evaluation, Molecular Docking and Pharmacokinetics

Danish Shahzad[1], Aamer Saeed [1,*], Fayaz Ali Larik [1], Pervaiz Ali Channar [1], Qamar Abbas [2], Mohamed F. Alajmi [3], M. Ifzan Arshad [1], Mauricio F. Erben [5], Mubashir Hassan [6], Hussain Raza [6], Sung-Yum Seo [6] and Hesham R. El-Seedi [4]

1 Department of Chemistry, Quaid-I-Azam University, Islamabad 45320, Pakistan.
2 Department of Physiology, University of Sindh, Jamshoro76080, Pakistan
3 Department of Pharmacognosy, College of Pharmacy, King Saud University, Riyadh 11451, Kingdom of Saudi Arabia
4 Pharmacognosy Group, Department of Medicinal Chemistry, Biomedical Center (BMC), Uppsala University, SE-751 23 Uppsala, Sweden
5 CEQUINOR (UNLP, CONICET-CCT La Plata), Departamento de Química, Facultad de Ciencias Exactas, Universidad Nacional de La Plata, Boulevard 120 e/60 y 64 N°1465, La Plata 1900, Argentina
6 Department of Biological Sciences, College of Natural Sciences, Kongju National University, 56 Gongjudehak-Ro, Gongju, Chungnam republic of Korea

Supplementary data

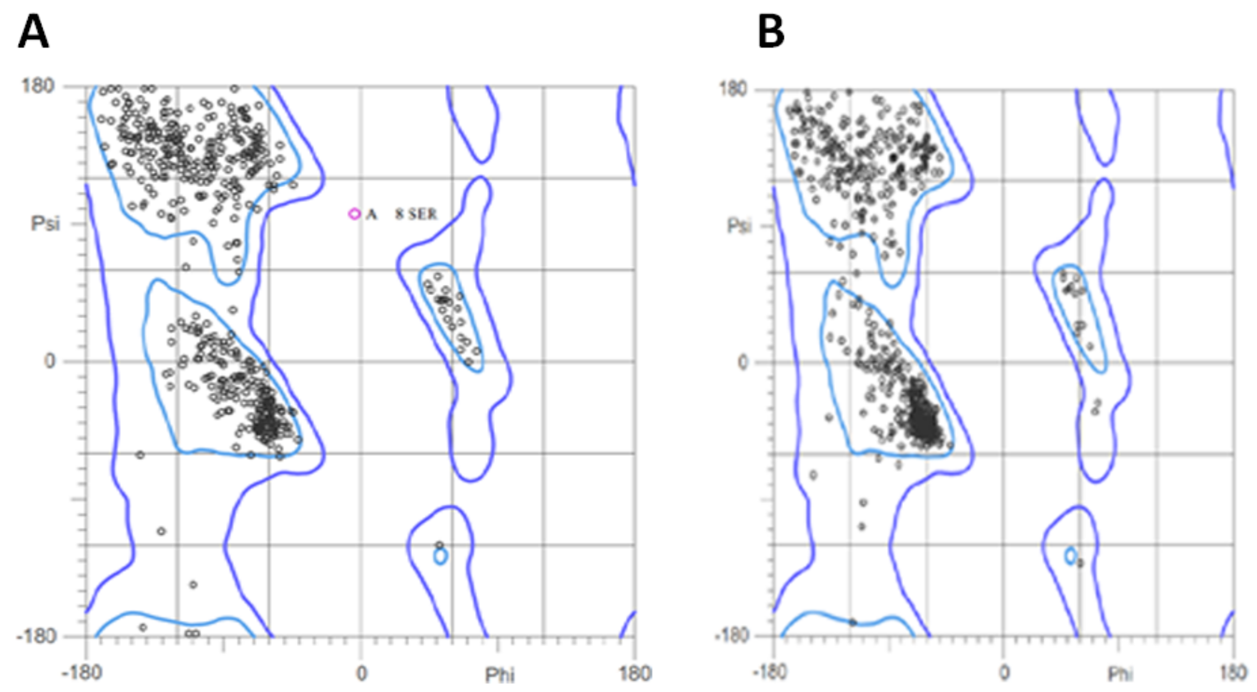

Figure. S1. Ramachandran graphs of α-amylase (A) and α-glucosidase (B)

Docking Energy value equation

$$\Delta G\ binding = \Delta Ggauss + \Delta Grepulsion + \Delta Ghbond + \Delta Ghydrophobic + \Delta Gtors \qquad \text{(i)}$$

Here, ΔG gauss: attractive term for dispersion of two gaussian functions, ΔGrepulsion: square of the distance if closer than a threshold value, ΔGhbond: ramp function - also used for interactions with metal ions, ΔGhydrophobic: ramp function, ΔGtors: proportional to the number of rotatable bonds.

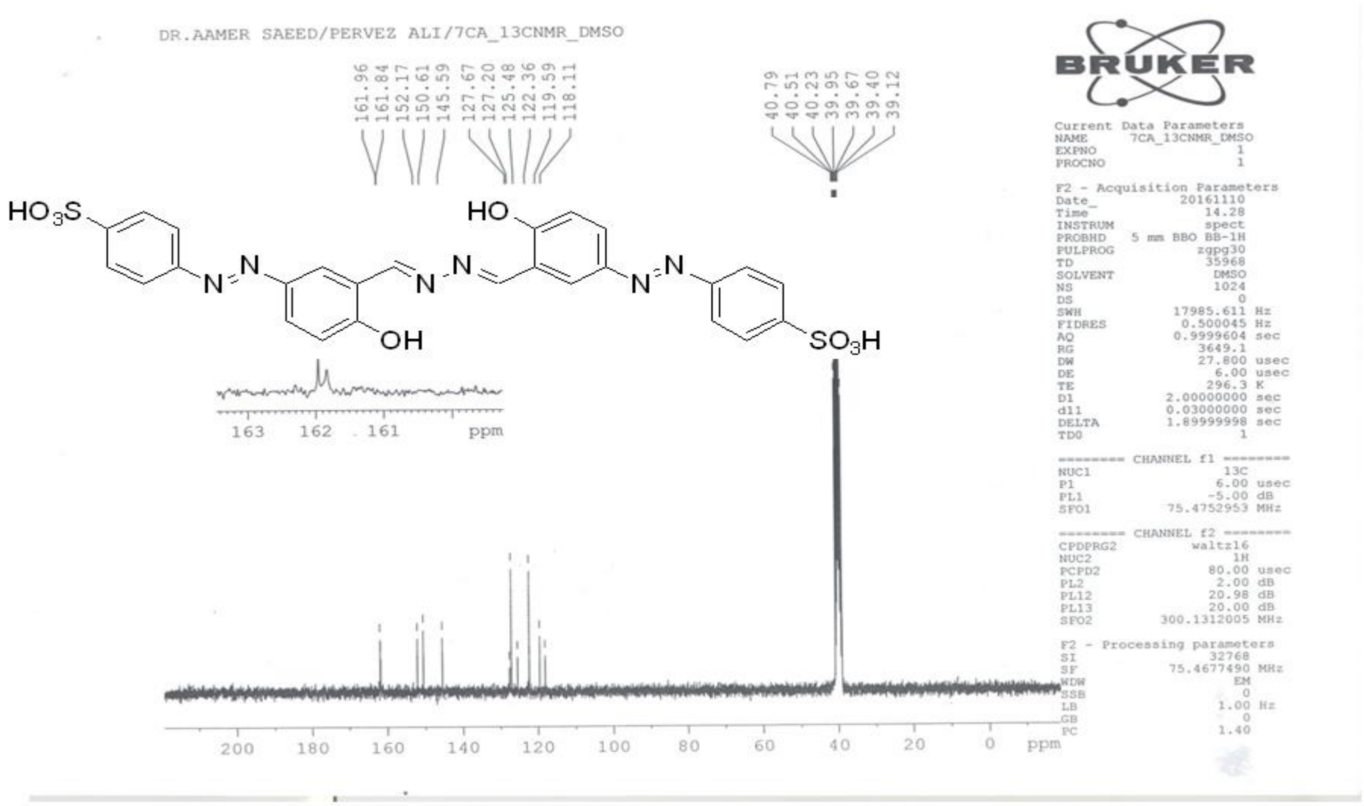

Figure –S2 ^{13}C NMR spectrum of (**5f**)

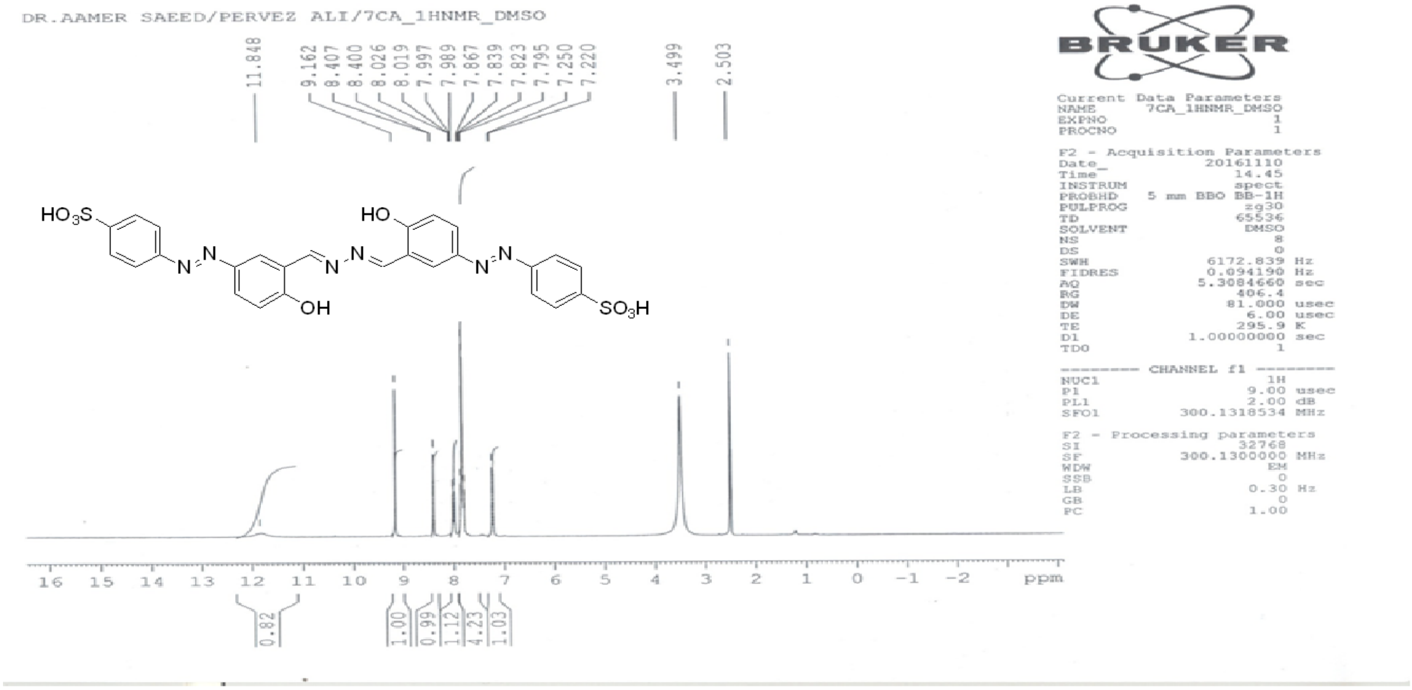

Figure –S3 ^{1}H NMR spectrum of (**5f**)

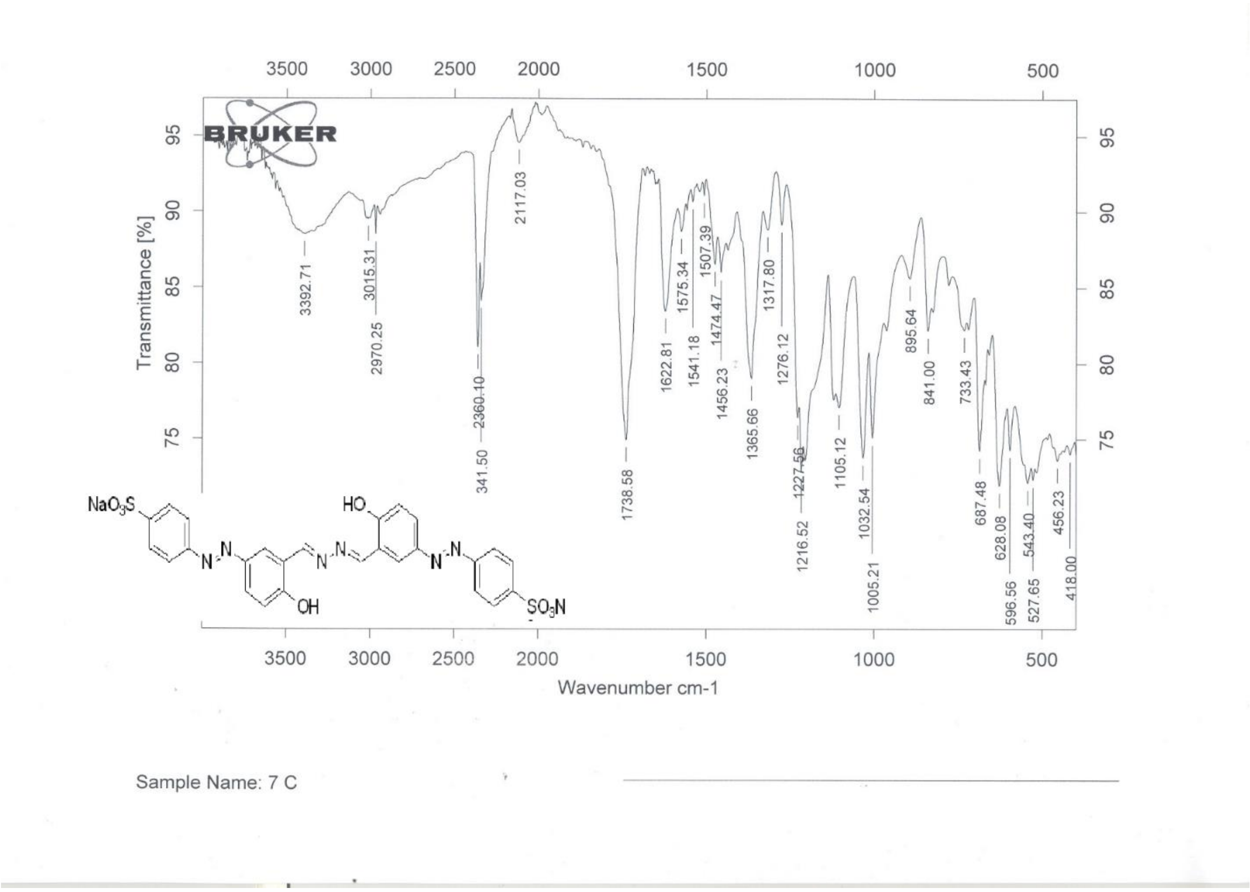

Figure S4. FT-IR spectrum of **5f**
